# The complete chloroplast genome sequences of the *Iris loczyi* kanitz (Iridaceae)

**DOI:** 10.1080/23802359.2020.1790312

**Published:** 2020-07-20

**Authors:** Tae-Young Choi, Seung-Hwan Oh, Chang-Gee Jang, Hae-Won Kim, Aleksey Kim, Soo-Rang Lee

**Affiliations:** a Forest Biodiversity Division, Korea National Arboretum, Pocheon, Republic of Korea; b Department of Biology Education, Kongju National University, Gongju, Republic of Korea

**Keywords:** *Iris loczyi*, complete chloroplast genome, Iridaceae

## Abstract

*Iris loczyi* is a perennial rhizomatous herb distributed in Central Asia. We examined genomic architecture of the complete chloroplast genome in *I. loczyi* by assembling the Illumina MiSeq reads using de novo strategy. The chloroplast genome is 150,940 bp in length harboring 79 protein-coding genes, 30 tRNA genes, and four rRNA genes. It exhibits typical quadripartite structure comprising LSC (80,907 bp), SSC (17,853 bp), and a pair of IRs (26,090 bp). Phylogenetic analysis of 20 chloroplast genomes from Asparagales revealed that Iridaceae is a monophyletic group and the *I. loczyi* is clustered together with the congener, *I. sanguinea.*

*Iris loczyi* Kanitz (Iridaceae), is a rhizomatous perennial herb distributed in Central Asia including Afghanistan, Iran, Kazakhstan, Kyrgyzstan, Pakistan, Tadzhikistan, Uzbekistan (Khassanov and Rakhimova 2012). Like most plants in *Iris* L., *I. loczyi* is a well- recognized plant for its economical values in pharmaceutical and horticultural practices (Crisan and Cantor 2016). The plant has recently drawn much attention as it contains various secondary metabolites that might have the potential to manage diabetes (Mosihuzzman et al. 2013). However, the genomic information applicable for breeding program and other biological studies is scarce. In the present study, we investigated the genomic architecture in the whole chloroplast genome of *I. loczyi* using whole genome shotgun sequencing.

We collected young leaves of *I. loczyi* from Issyk kul, Kyrgyzstan (N42°47’0.8″, E77°31′41.9″). The voucher specimen was prepared and deposited at the Herbarium of Korea National Arboretum (KH) with the accession number KHB1544459. The total genomic DNA was extracted followed by manufacturer’s protocol (Quiagen, Hilden, Germany). After library preparation, the prepared libraries were sequenced on Illumina MiSeq platform (Illumina, San Diego, CA). Eight million high-quality 300 bp paired-end reads were obtained. We assembled 2.85 GB reads with *de novo* strategy using CLC Assembly Cell package (ver. 4.2.1) followed by Kim et al. (2015). The genes were predicted with GeSeq (Tillich et al. 2017) and manually curated based on Blast search result. The simple sequence repeats were investigated with MISA (Beier et al. 2017).

The complete chloroplast genome of *I. loczyi* (MT254070) is 150,940 bp in length with the typical quadripartite structure comprising LSC (80,907 bp), SSC (17,853 bp), and a pair of IRs (26,090 bp). The cp genome contained 113 genes including 79 protein-coding genes, 30 tRNA genes, and four rRNA genes. 463 simple sequence repeats were identified in the cp genome, most of which was penta-nucleotide.

To investigate its phylogenetic relationship, the entire chloroplast genome sequences of 20 Asparagales taxa were aligned in MAFT (Katoh et al. 2019). All sequences other than *I. loczyi* were downloaded from NCBI Genebank. We assigned *Disporum sessile* D.Don (Colchicaceae) as an outgroup following phylogenetic relationships based on APG system (Stevens 2017). We inferred the phylogeny using Maximum-likelihood algorithm implemented in RAxML v. 4.0 with GTR GAMMA model. For the clade support, 1000 bootstrap replicates were used. The five species of Iridaceae formed a monophyletic group (BP = 100) with strong support on ML tree (Figure 1). In ML tree, Iridaceae grouped together with Asparagaceae, Amarylidaceae and Asphodelaceae, while Orchidaceae and Asteliaceae formed separate clades respectively. The ML tree also indicated that *I. loczyi* is closely related with *I. sanguinea* which is consistent with the previous subgeneric classification (Wilson 2004).

**Figure 1.**
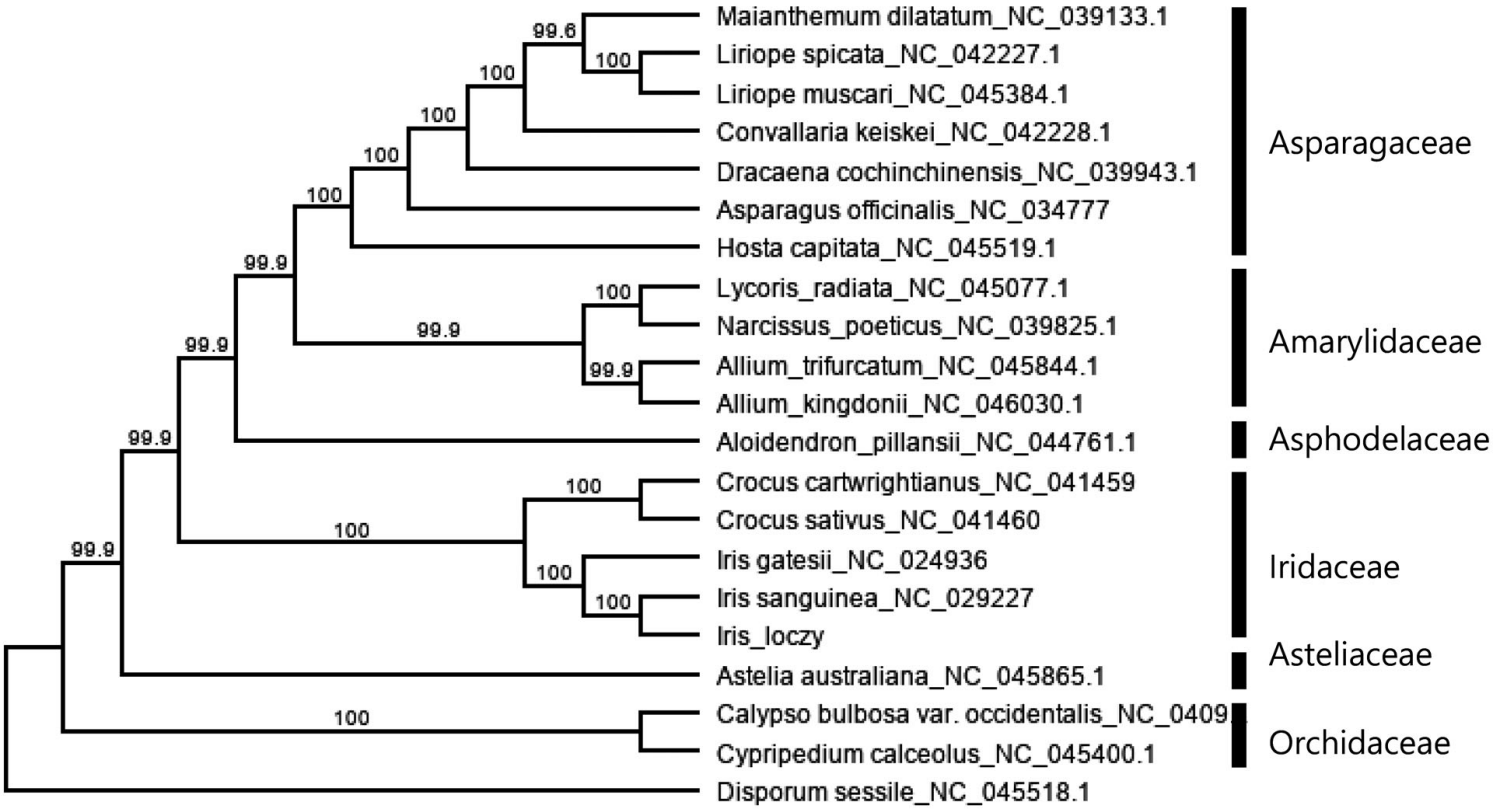
Maximum-likelihood (ML) tree based on chloroplast genome sequences of 20 species of Asparagales, numbers on the nodes indicated the bootstrap support value (>50%).

## Data Availability

The data that support the findings of this study are openly available in NCBI GenBank at https://www.ncbi.nlm.nih.gov/genbank/, accession number MT254070.
